# Cleft missions of the Korean Association of Maxillofacial Plastic and Reconstructive Surgeons

**DOI:** 10.1186/s40902-019-0219-z

**Published:** 2019-09-24

**Authors:** Young-Wook Park

**Affiliations:** Department of Oral and Maxillofacial Surgery, College of Dentistry, Gangneung-Wonju National University, Gangneung, Republic of Korea

Cleft lip and/or palate (CLP) is a congenital oro-facial anomaly, which has contributed to the significant disease burden, especially in developing countries. To overcome the global disease burden, humanitarian international missions for CLP in developing countries are essential. Since 1996, the Korean Association of Maxillofacial Plastic and Reconstructive Surgeons (KAMPRS) has performed voluntary surgical missions for CLP in China, Kazakhstan, and Vietnam. Now, I would like to focus on the friendship operation in Vietnam consisting of 15 visits between 2004 and 2018.

Why Vietnam? Korea and Vietnam suffered from a similar war in modern history. Therefore, the people of the two countries share emotional empathy. Additionally, Korean people owe the Vietnamese due to the overseas dispatch of Korean forces at the end of the Vietnam War. So, we could have a good surgeon-patients’ rapport as well as a good relationship with the local staffs. Another reason is that CLP is relatively common and suspected to be endemic in Vietnam and believed to result from the scattering of defoliants including dioxin [1]. Lastly, such activities had partly contributed to the evolution of CLP care in KAMPRS as Dr. Millard developed his famous rotation-advancement method from his Korean experience immediately after the Korean War.

Our partner is the National Hospital of Odonto-Stomatology (NHOS) in Hanoi. They have recruited patients for the cleft mission. In 2004, our honorary professor Byong-Il Min first visited the hospital for planning and setting our mission. Since then, lots of oral and maxillofacial faculties have participated in this activity. On the 10th anniversary, we changed the project name from “Charity Operation” to “Friendship Operation” that means we are companion. Besides the free-of-charge surgical care, we tried all our best to educate local doctors for CLP care and to have a program of personal exchange for mutual trust.

KAMPRS team of volunteers usually consists of 15 oral and maxillofacial surgeons and a Vietnamese translator. Travel fees are their own expenses and partly from donations by the members of KAMPRS. Vietnamese anesthesiologists and nurses work together during the friendship operation. Annually, our mission is scheduled for a week. The standard activities are initiated just after arrival. We screen the patients to select the candidate for operation and set the plan for operations. After 5 days of surgery, we finally check the patients who received operations with Vietnamese staffs. After coming back to Korea, we follow up postoperative status of the patients through communication with the Vietnamese staffs.

In 15 missions, a total of 478 operations had been performed, consisting of primary cheiloplasty and palatoplasty, secondary revisions, and operations for patients with facial cleft. Swanson reported half of the Vietnamese children were not able to access a surgical cleft care prior to 18 months of age [2]. In our patients’ group, the primary cheiloplasty was performed at the age of 13 months, and the primary palatoplasty was done at the age of approximately 6 years on average. That is because primary and older patients had the priority in our selection of candidate patients. Most of the patients were past the appropriate time of surgery, and sometimes, very old patients were shown up.

In friend operation, patients never underwent any type of preoperative hospital care. Consequentially, the deformities are exaggerated. So, our surgical strategies were as follows: For repair of a cleft lip, we performed a robust orbicularis muscular reconstruction. Nasolabial repair with concomitant premaxillary setback was performed in case of a bilateral deformity. Two-flap palatoplasty with a pedicled buccal fat pad was performed to decrease the fistula rate [3]. Delayed alveolar bone graft was done when indicated. Active nasal correction was performed because there is no guarantee that these patients will have additional surgery in their future life.

As far as the author’s best knowledge, more than half of cleft surgery are assumed to be performed in mission environments all over the world. Therefore, we need to optimize the CLP care for these patients. In the friendship operation of KAMPRS, there was minimal morbidity and no mortality, and top-quality surgical services have been provided. In conclusion, although the ultimate goal for a global cleft mission is to establish an institute providing long-term cleft cares, the voluntary surgical mission of KAMPRS still have an important role in lessening the disease burden of Vietnam.

## References

[1] Natsume N, Kawai T, Le H (1998). In Vietnam, many congenital anomalies are believed to result from the sacttering of defoliants, including dioxin. Cleft Palate Craniofac J.

[2] Swanson JW, Yao CA, Auslander A, Wipfli H, Nguyen TH, Hatcher K (2017). Patient barriers to accessing surgical cleft care in Vietnam: a multi-site, cross-sectional outcomes study. World J Surg.

[3] Kim MK, Han W, Kim SG (2017). The use of the buccal fat pad flap for oral reconstruction. Maxillofac Plast Reconstr Surg.

